# Diurnal rhythms in melatonin and cortisol in very preterm human milk

**DOI:** 10.3389/fnut.2026.1845043

**Published:** 2026-06-16

**Authors:** Demy van Gilst, Jorine A. Roelants, Alja Bijlsma, Irwin K. M. Reiss, Koen F. M. Joosten, Jeroen Dudink, Marijn J. Vermeulen, Inês Chaves

**Affiliations:** 1 Department of Molecular Genetics, Erasmus University Medical Centre, Rotterdam, Netherlands; 2 Department of Neonatology, Wilhelmina Children's Hospital, University Medical Center Utrecht, Utrecht University, Utrecht, Netherlands; 3 Division Preventive Youth Health Care, Public Health Services Region Utrecht (GGD Regio Utrecht), Utrecht, Netherlands; 4 Department of Neonatal and Pediatric Intensive Care, Division of Neonatology, Erasmus MC Sophia Children’s Hospital, Erasmus University Medical Center, Rotterdam, Netherlands; 5 Department of Neonatal and Pediatric Intensive Care, Division of Pediatric Intensive Care, Erasmus MC Sophia Children’s Hospital, University Medical Center Rotterdam, Rotterdam, Netherlands

**Keywords:** chrononutrition, cortisol, diurnal rhythm, human milk, lactation, melatonin, nutrition, preterm infants

## Abstract

**Introduction:**

Human milk (HM) contains bioactive compounds and hormones vital for infant neuro- and circadian-development. While healthy term-born infants receive time-of-day cues naturally through breastfeeding, preterm infants in the Neonatal Intensive Care Unit (NICU) are typically fed with pooled expressed milk via tube feeding, which lacks time-of-day specificity. Despite growing interest in chrononutrition and circadian entrainment in the NICU, little is known about diurnal hormonal rhythms in preterm HM and their potential role in circadian development.

**Methods:**

We investigated diurnal rhythmicity of cortisol and melatonin in human milk from mothers of very preterm infants (born before 30 weeks gestational age) across different lactational stages. Milk series consisted of 13 to 17 (median 15) samples, collected over three consecutive days. Cortisol and melatonin concentrations were measured using enzyme-linked immunosorbent assays (ELISA) and rhythmicity was assessed using non-linear cosine curve fitting.

**Results:**

In total, 25 milk series were included. Melatonin exhibited a clear diurnal rhythm in all 25 HM series, while cortisol showed rhythmicity in 18 out of 25 HM series. The mean peak time for melatonin occurred at 03:58 (AM) ± 1.46 h; for cortisol, the mean peak time occurred at 08:29 (AM) ± 1.86 h. There was no significant association between lactational stage and peak time or amplitude for either hormone. In a preliminary subgroup analysis, milk from mothers of female infants showed a significantly earlier cortisol peak compared to mothers of male infants (*p* < 0.01).

**Conclusion:**

Despite the immaturity of the lactational system after very preterm birth, consistent diurnal variations in cortisol and melatonin were present across all lactational stages. These findings demonstrate that maternal hormonal rhythmicity is preserved in human milk from the earliest stages of preterm lactation, providing a biological rationale for further research into whether time-of-day matched feeding strategies may provide biologically relevant temporal signals that warrant investigation regarding their potential role in circadian entrainment in very preterm infants.

## 1 Introduction

Human milk (HM) is an essential source of nutrition for infants, supporting optimal growth and development by providing a complex mixture of nutrients, hormones and bioactive components (1–4). Beyond providing micro- and macronutrients, human milk also plays a role in neurodevelopment, development of the infant’s immune system, and microbiome by providing essential growth factors, immunoglobulins and cytokines (1, 3). To meet infants’ individual needs, HM composition is highly dynamic and influenced by infant, maternal and environmental factors (1, 5–8). The composition also varies between term-born and preterm born infants. Preterm milk is specifically tailored to address the unique requirements of premature infants (9–14). It has also been shown that the composition of term milk changes during a feeding, throughout the day and between lactational stages (1, 2, 15–17).

The circadian system is responsible for daily anticipation of environmental changes by generating near 24-h rhythms in behavior and physiology (18, 19). The mammalian circadian system consists of a central clock in the suprachiasmatic nucleus (SCN) and peripheral clocks in nearly every cell of the body (18, 19). Under normal conditions, these rhythms are synchronized daily by environmental cues, endocrine signaling and autonomic innervation. This synchronization ensures that physiological functions, such as sleep–wake cycles, metabolic regulation and hormone production, will occur at the appropriate time of day (18, 19). At the molecular level, circadian oscillations are driven by autoregulatory feedback loops of transcription and translation of core clock genes, generating an oscillation with a near 24-h period (18).

In both circadian and lactational programming, cortisol and melatonin are key hormones. Cortisol, the final hormone produced by the hypothalamic–pituitary–adrenal (HPA) axis, regulates stress responses and follows a diurnal rhythm, peaking in the morning (20, 21). In addition, maternal glucocorticoids (GCs) play an important role in lactogenesis, mammary gland differentiation and milk secretion (21, 22). Melatonin is a neuro-hormone synthesized by the pineal gland and is known to regulate various processes. It regulates sleep by promoting sleepiness and is also considered a potent antioxidant (23–25). Melatonin production is primarily driven by the SCN in response to decreases in light, and therefore peaks at night (25). Both cortisol and melatonin are known to be important for circadian regulation. Previous literature showed that cortisol and melatonin are present in human milk and show a diurnal rhythm. This presence has been suggested to support development of the circadian system in infants (8, 17, 22, 26–29).

This synchronization is particularly important for infants born very preterm, as their endogenous circadian rhythms have not yet been established, and typically develop only weeks to months after birth (30, 31). Infants born before 30 weeks GA exhibit pronounced immaturity of multiple organ systems, including the central nervous system and the circadian system (4). At this age, endogenous SCN activity is still immature and pineal melatonin production is absent. Consequently, these infants are more reliant on maternal derived circadian cues, including those transmitted through human milk, to support circadian development (31, 32). Importantly, mammary gland and milk production are at an early stage in the event of a very preterm delivery, and any rhythms identified in milk composition are likely present later as well (15). The maturation of the circadian system is likely also influenced by the lack of strict rhythmicity in natural light–dark cycles, frequent, often mistimed feedings and medical interventions in the Neonatal Intensive Care Unit (NICU) where preterm infants are admitted after birth (33, 34). In clinical practice, expressed milk is frequently stored and administered hours after expression, often without consideration of the time of day at which it was expressed. Reported storage intervals typically range from several hours to more than 24 h, and donor milk (DM) from multiple mothers and collection times is routinely pooled, thereby eliminating intrinsic diurnal variation (35). Consequently, a substantial portion of feedings provided to preterm infants may be temporally mismatched relative to the maternal circadian phase.

Extensive literature in animals and human adults shows that disruption of the circadian rhythm is associated with a wide range of negative health outcomes, including cardiovascular diseases, metabolic disorders and impaired functioning of the immune system (36–41). Recently, the importance of the timing of nutritional intake in relation to the body’s biological rhythms has triggered growing interest in the concept of chrononutrition (42, 43). Chrononutrition is defined as the interaction between body clocks, metabolism and meal timing. HM may play a role in transmitting maternal timing cues to the infant, thereby potentially influencing the infant’s circadian rhythms (2, 15, 26, 44). The presence of melatonin and cortisol in term-HM is thought to provide time-of-day cues that help synchronize the internal clock with environmental light–dark cycles (27, 45). Knowledge on diurnal variation in human milk is particularly relevant for optimizing clinical practice in the NICU. If human milk can serve as an entrainer of the neonatal circadian rhythm, aligning feeding schedules with maternal circadian phase may represent a future strategy to support circadian development in the NICU.

In the present study, we characterize the presence of diurnal rhythms in cortisol and melatonin in very preterm human milk. To date, high-frequency, multi-day individual hormonal rhythms in (very) preterm HM have not been previously described. Characterizing whether maternal hormonal rhythms are present in milk after very preterm delivery is a necessary first step before functional studies can address whether these signals contribute to circadian entrainment in preterm infants.

## 2 Materials and methods

### 2.1 Participants

This retrospective cohort study was conducted in lactating mothers whose infants were admitted to the NICU of the Erasmus MC-Sophia Children’s Hospital, Rotterdam, Netherlands, between January 2017 and November 2024. The study was approved by the institutional review board (number MEC-2017-1175) and all participants provided informed consent. The inclusion criteria consisted of (1) preterm delivery before 30 weeks gestational age (GA), (2) availability of 13 or more HM samples of at least 10 mL each, collected over 72 consecutive hours, corresponding to a minimum of four samples per day; and (3) samples not required for clinical feeding purposes. Participants who were unable to give informed consent (e.g., due to a language barrier) and maternal age <18 years were not considered eligible.

### 2.2 Data collection

Baseline characteristics were retrospectively extracted from electronic medical files. Maternal characteristics included parity, mode of delivery, medical history and prolonged rupture of membranes (>24 h before birth). Neonatal characteristics included gestational age, birth weight, infant sex and survival status. Missing data from patient baseline characteristics were excluded from analysis. Milk samples were categorized by lactational stage: colostrum (milk collected from birth (day zero) up to the fourth day of life), transitional (milk collected from day five up to the second week postpartum) or mature (collected from 2 weeks postpartum onwards) (1).

### 2.3 Human milk sample collection

Milk was expressed following local guidelines, using a professional double-sided electronic breast pump (Medela Symphony, Medela AG, Baar Switzerland). In practice, mothers typically expressed milk five to eight times per day, reflecting standard clinical recommendations for maintaining lactation during NICU admission. Due to the retrospective study design, the exact timing of expression was determined by the mother and was not standardized to fixed time points. Milk samples were labeled according to the hospital protocol (name, date and time of expression). Only samples collected and labeled in accordance with hospital regulations for clinical care and stored in the hospital freezer (at −20 °C) were eligible for inclusion. Samples were only used for this study if they were definitively not needed for clinical feeding of the infant. As few lactating mothers have abundant milk when their infant survives NICU stay, most series were collected from mothers whose infant deceased during NICU stay and donated their milk for research purposes. Following inclusion, milk samples were aliquoted in microtubes (Micronic, Lelystad, Netherlands) and subsequently stored in dark at −80 °C until thawed for analysis. In total, milk samples underwent two freeze–thaw cycles during the study process. Duration between milk expression and analysis varied between 1 and 5 years, depending on the year of inclusion. During aliquoting and hormone quantification, the samples were handled under standard laboratory lighting and not specifically protected from light.

### 2.4 Quantification of melatonin levels

Melatonin levels were measured using Melatonin Direct Saliva ELISA kit (Kit RE54041, IBL International, Hamburg, Germany) according to the manufacturer’s instructions. The protocol included specific instructions for human milk dilutions and measurements. Prior to analysis, each milk sample was defrosted at room temperature and centrifuged at 13000 rpm for 20 min at 4 °C to obtain the skim milk fraction (aqueous phase). All skim milk samples were analyzed in duplicate and absorbance was measured with a spectrophotometer (SpectraMax iD3, Molecular Devices LLC, San Jose CA) at 450 nanometer (nm). Results were calculated according to a standard curve using 4 Parameter Logistics and expressed in pg./mL. If sample values were greater than 50 pg./mL, the sample was diluted 1:10 with Standard A and remeasured. Measured results were multiplied by the dilution factor to obtain the correct values. The limit of quantification was 0.854 pg./mL. The coefficients of variation were 13.9% (range 13.2–14.4%) and 18.4% (range 16.5–19.7%) for intra- and inter-assay, respectively.

### 2.5 Quantification of cortisol levels

Cortisol levels were measured using Cortisol free in Saliva ELISA kit (Kit RE52611, IBL International, Hamburg, Germany) according to the manufacturer’s instructions. The protocol included specific instructions for human milk dilutions and measurements. Prior to analysis, each milk sample was defrosted at room temperature and centrifuged at 13000 rpm for 20 min at 4 °C to obtain the skim milk fraction (aqueous phase). All skim milk samples were analyzed in duplicate and absorbance was measured with a spectrophotometer (SpectraMax iD3, Molecular Devices LLC, San Jose CA) at 450 nm. Results were calculated according to a standard curve using 4 Parameter Logistics and expressed in μg/dL. If sample values were greater than 2.2 μg/dL, the sample was diluted 1:10 with Standard A and remeasured. Measured results were multiplied by the dilution factor to obtain the correct values. The limit of quantification was 0.005 μg/dL. The intra-assay variation ranged from 3.2 to 6.1% and inter-assay variation ranged from 10.1 to 19.5%.

### 2.6 Statistical analysis

Descriptive key statistics, number (percentage), median [interquartile range (IQR), minimum-maximum] or mean [standard deviation (SD)] were used to describe baseline data. Rhythmicity in cortisol and melatonin concentrations was assessed in each individual series using a non-linear curve model in R statistical software (version 4.5.1) and R-package CircaCompare version 0.1.0 (46). In brief, this method fits a cosine curve with a fixed 24-h period to each individual hormonal time series and estimates three key parameters: the mesor (mean concentration around which the rhythm oscillates), the amplitude (half the peak-to-trough difference, reflecting the strength of the rhythm), and the acrophase or peak time (the time at which the fitted curve reaches its maximum). A series was considered a significant 24-h rhythm when the cosine fit yielded a *p*-value of <0.05. Graphical curve fits were constructed using R statistical software (version 4.5.1) with each dot representing one milk sample consisting of two individual measurements. In a single graph, cortisol and melatonin concentrations collected over three consecutive days are shown on a 24-h scale. All other statistical calculations and graphical visualizations were performed using Graphpad Prism (Graphpad V9.0, San Diego, CA) and SPSS Version 28.0 (IBM SPSS Statistics, Chicago, IL). A two-tailed *p* < 0.05 was considered statistically significant.

## 3 Results

### 3.1 Study population

**Figure 1:**
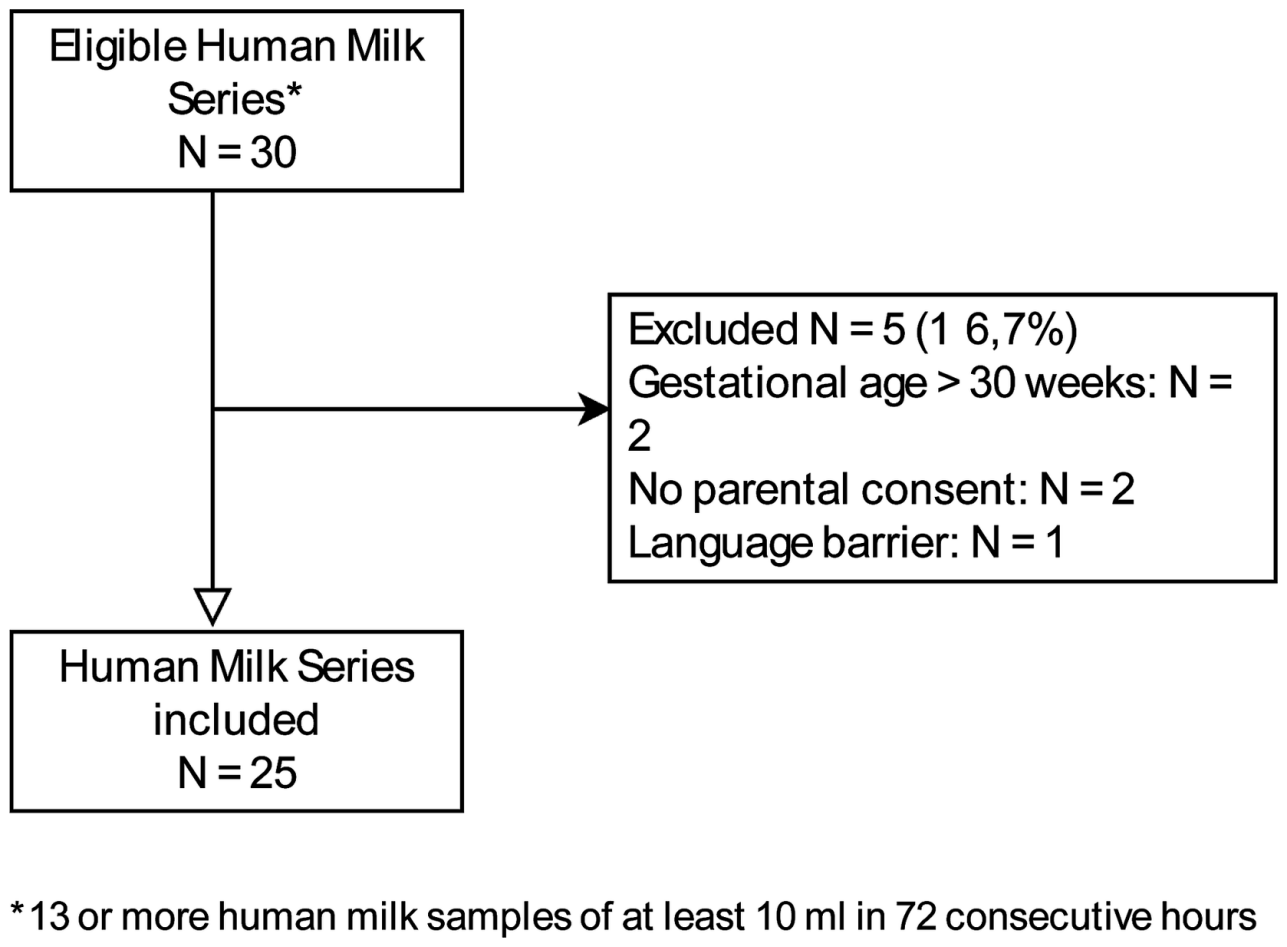
Inclusion flowchart.

A total of 30 milk series were eligible for inclusion. Of these, 5 series were excluded due to missing informed consent, GA > 30 weeks or a parental language barrier, leaving 25 series for analysis (Figure 1). Baseline characteristics of the analyzed cohort are presented in Table 1. The median [interquartile range (IQR)] gestational age at birth was 26 weeks (25 + 1–28 + 1), and the median birth weight was 800 g (650–975). Most infants were born via caesarean section (68%) and infant sex was evenly distributed (52% male, 48% female). Maternal corticosteroids were administered to 24 out of 25 mothers before delivery. A total of 23 infants (92%) died during NICU stay, with a median time of death occurring on day 8 (IQR: 7–15) postpartum. Causes of death included necrotizing enterocolitis/sepsis (52.2%), respiratory failure (21.7%), refractory shock (8.7%) and other causes (17.4%).

**Table 1:** Baseline participant characteristics.

Characteristics	*n* = 25
Maternal characteristics	
Hypertension	4 (16)
(gestational) DM	0 (0)
PPROM	5 (20)
Multiparity	8 (32)
Antenatal corticosteroids	24 (96)
Infant characteristics	
Pregnancy duration at birth (weeks^+days^)	26^+0^ [25^+0^;28^+1^]
Birth before 28 weeks GA	18 (72)
Birth weight (grams)	800 [650;975]
Birth weight SD score	0.10 [−0.71;0.75]
Sex assigned at birth (female)	12 (48)
Cesarean section	17 (68)
Deceased during NICU stay	23 (92)
Milk characteristics	
Median samples per series	15 [13;17]
Day of first milk sample	4 [3.5;9.5]
Duration of series (hours)	70 [65;72]

Data are presented as median [interquartile range] or *n* (%).

DM, diabetes mellitus; GA, gestational age; NICU, neonatal intensive care unit; *n*, number; PPROM, preterm prolonged rupture of membranes (>24 h before birth); SD score, standard deviation score.

### 3.2 Identification of diurnal rhythms in melatonin and cortisol in preterm human milk

**Figure 2:**
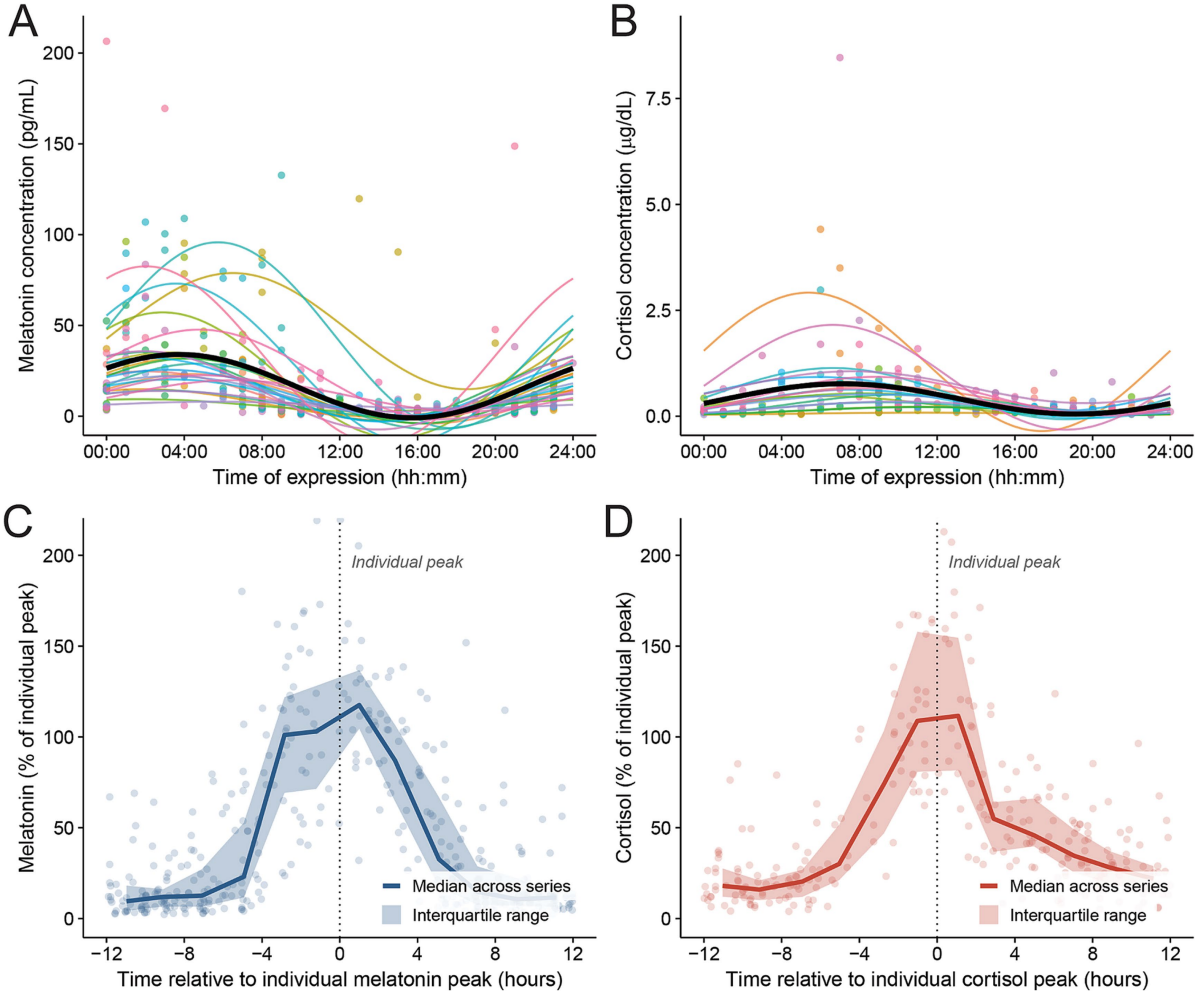
Significant 24-h rhythmicity and inter-individual variability in melatonin and cortisol levels in preterm human milk. **(A)** Melatonin cosinor fits for each individual milk series exhibiting diurnal rhythmicity (*n* = 25). **(B)** Cortisol cosinor fits for each individual milk series exhibiting diurnal rhythmicity (*n* = 18). The y-axis represents the melatonin (pg/mL) and cortisol (μg/dL) concentration, the x-axis represents time of expression (hh:mm). Each data point represents one milk sample. Each figure shows hormone concentrations from samples collected over three consecutive days on a 24-h scale. Each colored line and corresponding dots represent one individual milk series. The black solid line represents the average nonlinear regression fit. **(C)** Melatonin concentrations expressed as a percentage of each milk series’ own peak melatonin value, plotted against time relative to each series’ melatonin peak (*t* = 0 h; *n* = 25 series). **(D)** Cortisol concentrations expressed as a percentage of each milk series’ own peak cortisol value, plotted against time relative to each series’ cortisol peak (*t* = 0 h; *n* = 18 series with significant cortisol rhythm). Each dot represents one milk sample. The solid line represents the median concentration across series. The shaded band represents the interquartile range (IQR). The dashed vertical line marks the individual hormonal peak (*t* = 0).

All 25 HM series exhibited a clear diurnal rhythm in melatonin concentrations, with levels increasing between approximately 20:00 h (PM) and 5:00 h (AM) and decreasing during the day (Figure 2A). The mean peak time occurred at 03:58 (AM) ± 01.46 h (Supplementary Figure 1), with a mean peak concentration of 65.72 ± 83.13 pg./mL and a mean trough concentration of 2.29 ± 1.11 pg./mL. A diurnal rhythm in cortisol levels was observed in 18 out of 25 HM series, with concentrations rising from around 02:00 h (AM) until approximately 10:00 h (AM) (Figure 2B). The mean peak time for cortisol occurred at 08:29 (AM) ± 1.86 h (Supplementary Figure 1). In milk series exhibiting a rhythm, the highest mean cortisol concentration was 2.12 ± 3.93 μg/dL, while the lowest was 0.08 ± 0.04 μg/dL. A sensitivity analysis restricted to milk series from mothers of deceased infants (*n* = 23 of 25) yielded results consistent with the main analysis, with melatonin rhythmicity detected in all 23 series (mean peak time 03:32 ± 1.48 h) and cortisol rhythmicity in all 16 included series (mean peak time 08:17 ± 1.91 h; Supplementary Figure 2). Normalized concentration profiles, in which each series was aligned to its own hormonal peak (*t* = 0) and concentrations expressed as a percentage of the individual peak value, are presented in Figures 2C,D. After normalization, both melatonin and cortisol profiles demonstrated consistent bell-shaped curves centered at *t* = 0, with the spread of individual data points around the median reflecting inter-individual variability in hormone amplitude rather than absence of a consistent rhythm. Together, these results demonstrate a consistent diurnal rhythm in melatonin across all preterm HM series and in cortisol in the majority of series, with noticeable inter-individual variability in peak timing.

**Figure 3:**
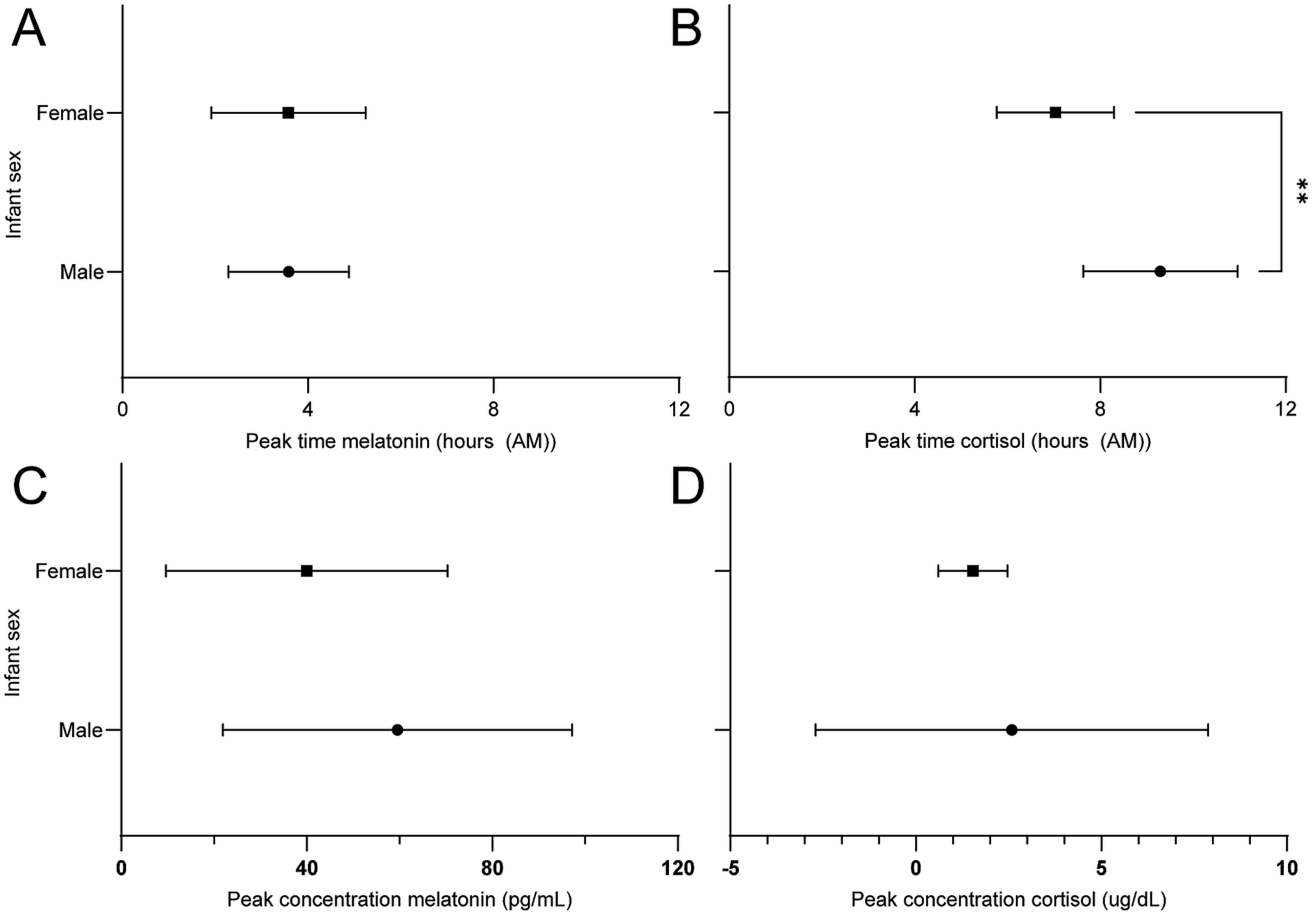
Melatonin and cortisol peak times and concentrations stratified by infant sex. **(A)** Peak time (hours) of melatonin stratified by infant sex (*n* = 12; female, *n* = 13; male). **(B)** Peak time (in hours) of cortisol stratified by infant sex (*n* = 8; female, *n* = 10; male). The peak time of cortisol was significantly earlier in mothers lactating female infants compared to those lactating male infants (*p* < 0.01). **(C)** Peak melatonin concentration (pg/mL) stratified by infant sex. **(D)** Peak cortisol concentration (μg/dL) stratified by infant sex. Data are presented as mean ± standard deviation (SD). Statistically significant differences are indicated as *p < 0.05; **p < 0.01.

For melatonin, no significant associations were found between lactational stage (Supplementary Figures 3A,C), gestational age (GA) (Supplementary Figures 4A,C), infant sex (Figures 3A,C) and peak time or melatonin concentration. Similar to melatonin, no significant association was found between the timing of the cortisol peak and lactational stage (Supplementary Figures 4B,D) or gestational age (Supplementary Figures 4B,D) (see Supplementary Tables 1, 2 for a full overview). Interestingly, milk from mothers of female infants showed a significantly earlier cortisol peak compared to milk from mothers of male infants (*p* < 0.01) (Figure 3B). In addition, cortisol concentrations in milk from mothers of male infants showed greater variability compared to milk intended for female infants (Figure 3D).

To assess the strength of the rhythms, the amplitude (i.e., the difference between peak and trough concentrations within a 24-h period) was calculated for each hormone. The mean amplitude observed for melatonin was 19.88 ± 16.05 pg./mL, while the mean amplitude for cortisol was 0.47 ± 0.65 ug/dL. The inter-individual variability was considerable for both hormones, with melatonin showing a broader range compared to cortisol. No significant associations were found between amplitude and gestational age, infant sex or lactational stage for both hormones (Supplementary Figures 5A–D). In addition, no correlation was observed between melatonin and cortisol amplitudes, indicating that the strength of the rhythms is independent from one another (data not shown).

### 3.3 Temporal alignment of melatonin and cortisol peak phases

**Figure 4:**
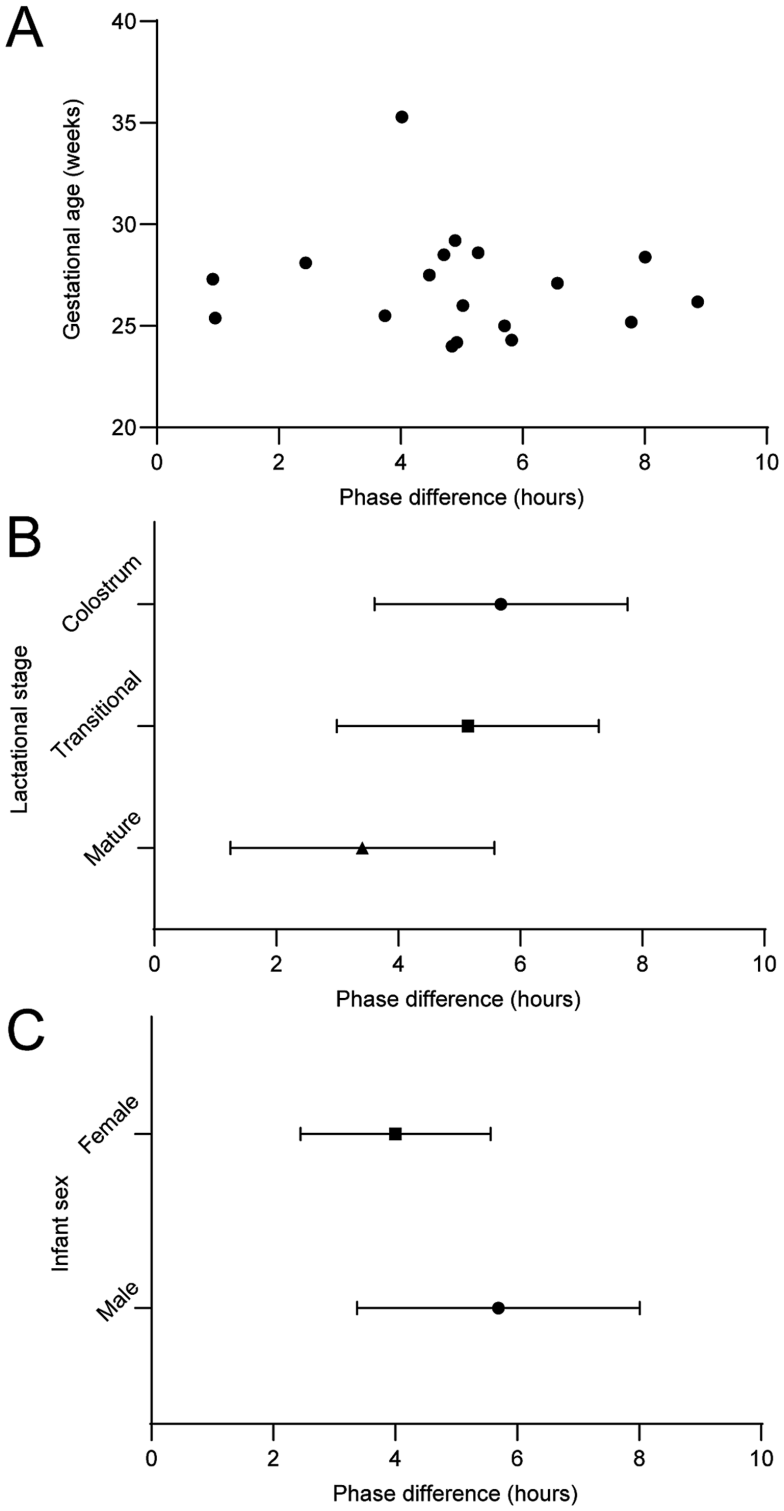
Phase differences between cortisol and melatonin. **(A)** Phase difference (in hours) according to gestational age (GA) at birth (weeks). **(B)** Phase difference according to lactational stage (colostrum, transitional, mature milk). **(C)** Phase difference stratified by infant sex. Data are presented as mean ± standard deviation (SD).

To explore the temporal relationship between circadian-regulated hormones in preterm HM, the phase difference between melatonin and cortisol was calculated. The phase difference was defined as the time in hours between the melatonin and cortisol peak within each milk series that showed a rhythm in both hormones (*n* = 18). The mean phase difference was 4.94 ± 2.14 h, indicating that melatonin levels consistently peaked several hours before cortisol. In addition, phases varied considerably between mothers, suggesting that the coordination between the rhythms differs per individual. No significant associations were found between the phase difference and gestational age or lactational stage (Figures 4A,B). Notably, although not statistically significant, milk from mothers of female infants tended to show a shorter phase difference than that from mothers of male infants (4.00 ± 1.56 vs. 5.69 ± 2.32, *p* = 0.097, respectively) (Figure 4C) (see Supplementary Tables 1, 2 for a full overview). These results suggest consistent temporal alignment of circadian hormones in preterm HM with known 24-h rhythms in both term HM and blood plasma.

## 4 Discussion

In the present study, our analysis of human milk from mothers of very preterm infants confirmed diurnal variations in both cortisol and melatonin across all lactational stages. Melatonin showed robust rhythms in all 25 HM series, while cortisol exhibited rhythmicity in 18 out of 25 series. A sensitivity analysis restricted to milk series from mothers of deceased infants was consistent with the main analysis, indicating that the observed diurnal rhythms are not explained by the survival status of the infant. No significant associations were found between hormone peak times, concentrations, and lactational stage or gestational age. Interestingly, we observed a significant difference in cortisol peak timing between male and female infants, a novel finding not previously described in literature.

Consistent with previous studies in term HM, and as hypothesized, we observed that nocturnal melatonin levels were higher than day-time levels (1, 2, 11, 27). Additionally, in most series, cortisol exhibited a robust diurnal rhythm, peaking early in the morning and reaching a trough around midnight. This observation is in agreement with other studies in term-born infants (1, 7, 47–49). However, previous studies examining daily variation in HM components relied on limited timepoints per day, typically comparing only a single morning and evening sample (2, 16, 50, 51). In our study, a median of five samples per day was collected over three consecutive days, enabling detection of individual 24-h rhythms and inter-individual variability that previous studies could not capture.

Although sex-specific differences in milk composition have been reported (52–55), differences in the timing of hormonal peak concentrations between mothers of male and female infants have not been previously described. Given the small subgroup sizes (*n* = 12 female, *n* = 13 male) and the exploratory nature of this analysis, this observation should be interpreted with substantial caution and considered preliminary. The biological mechanisms that could underlie sex-specific timing of hormonal rhythms in human milk remain unclear, and dedicated, adequately powered prospective studies are required to determine whether this finding is reproducible and, if so, whether it carries functional implications for the infant.

A noteworthy aspect of our findings is the preservation of maternal hormonal rhythmicity despite the exceptionally challenging circumstances of very preterm birth and prolonged NICU hospitalization. The studied cohort likely experienced high levels of psychological distress, sleep disruption and medical interventions, factors known to perturb maternal circadian physiology. The detection of a diurnal melatonin rhythm in all 25 series, and of a cortisol rhythm in the majority of series, suggests that maternal circadian signaling during early lactation is biologically resilient. Independent of any downstream effects on the infant, this resilience is itself a meaningful observation, indicating that human milk continues to carry temporal biological information even under conditions in which maternal rhythmicity could plausibly have been disrupted.

Beyond the diurnal rhythms themselves, we observed phase differences (i.e., differences in hormonal peak time) between mothers, suggesting variations in circadian phase that may reflect maternal chronotype or environmental and behavioral factors. Generally, chronotypes vary from early to late and influence the timing of hormone secretion in blood (56, 57), and this is thought to be mirrored in human milk: morning-type mothers show earlier melatonin and cortisol peaks, while evening-type mothers show later peaks (58–60). In our cohort, melatonin peaks ranged from approximately 01:00 to 06:00 a.m., and cortisol peaks ranged from around 06:00 to 11:00 a.m. This distribution closely mirrors the variability seen in adult blood across the general population (23). Together, these findings support the hypothesis that maternal chronotype plays a role in hormonal secretion in human milk. Furthermore, our findings suggest that, despite preterm delivery, maternal rhythms are still present.

In healthy full-term infants, maternal melatonin is an essential source during the first 3 months of life (1, 8, 27, 45). Endogenous melatonin rhythms emerge around 9–12 weeks after birth and continue to develop over the first 6 months of life (31). During this period, breast milk provides an external source of melatonin that may contribute to circadian alignment and development. When breastfed at home, the concept of chrononutrition is inherently applied, as breastfeeding typically aligns with the mother’s natural rhythms. In this context, chrononutrition occurs naturally, ensuring appropriate synchronization and further promoting optimal circadian alignment of the infant.

In contrast, when an infant is born very preterm and admitted to the NICU, a substantial mismatch with the uterine environment arises (33, 34, 61, 62). The infant loses maternal entrainment cues normally received in utero (33). In addition, NICU environments often lack strict physiological rhythmicity in light exposure and feeding time, in stark contrast to the regular routines at home. More importantly, these infants are often fed with pooled (donor) human milk, which combines milk expressed at different times of the day (26, 34, 44). This practice leads to the loss of the natural diurnal variation in HM components. In addition, donor milk (DM) use in the NICU does not take diurnal rhythms into account (4). DM is submitted to Holder pasteurization (HoP) to ensure microbiological safety in human milk banks (63). HoP is known to affect the concentration of several bioactive compounds, including melatonin (64, 65). In contrast, cortisol is relatively thermostable and is likely not affected by HoP (66). It has been hypothesized that the absence of these time-specific signals could affect the development of a robust diurnal rhythm, and, in turn sleep, metabolic processes and immune function in preterm infants (22).

### 4.1 Limitations

Several limitations must be considered when interpreting these results. First, the study cohort has an unusual composition: 23 of 25 (92%) died during NICU stay, and most milk series were therefore collected from mothers in extraordinarily stressful conditions including prolonged hospitalization, sleep disruption and grief. Such circumstances are known to activate the maternal HPA-axis and could plausibly influence circulating cortisol concentrations, sleep–wake organization and lactation physiology (67). All of these factors may in turn affect the hormonal composition of expressed milk. Although our sensitivity analysis restricted to the deceased-infant series yielded results consistent with the main analysis, this does not exclude the possibility that maternal stress influenced absolute hormone concentrations contributed to the absence of rhythmicity in some series or shaped inter-individual variability in peak timing and amplitude. These findings should therefore be considered representative of this specific clinical context, and their generalizability to the broader preterm population needs to be confirmed in future studies. Nevertheless, the detection of a consistent diurnal rhythm in 18 of 25 milk series suggests that maternal hormonal rhythmicity in milk is relatively resilient to acute stress. Second, the absence of detailed maternal data including light exposure, sleep–wake patterns, nutritional intake, and physical activity limits our ability to characterize the external determinants of hormonal rhythm timing (6). Future studies should incorporate maternal actigraphy and chronotype assessment alongside standardized expression times to fully characterize individual hormonal rhythms and phase characterizations.

Third, the potential influence of antenatal corticosteroid administration (administered to 24 out of 25 mothers) cannot be fully excluded. This could possibly lead to suppression of the maternal HPA-axis and therefore diminish the effect of a maternal cortisol rhythm. However, the short treatment course is unlikely to cause sustained HPA-axis suppression (68). Fourth, two methodological aspects of the melatonin measurements warrant explicit discussion. Melatonin is light-sensitive, but our samples were aliquoted and quantified under standard laboratory lighting conditions. In addition, storage duration between expression and analysis varied widely. These conditions may have affected the absolute melatonin concentrations. Nevertheless, the consistent detection of robust diurnal rhythms across all 25 series indicates that the relative within-series differences were preserved. Finally, while the detection of melatonin rhythmicity in all 25 series underscores the robustness of the sampling strategy, the relatively small overall sample size (although comparable to other studies on human milk (16, 50, 51)), limits the statistical power for subgroup analyses, and findings related to infant sex in particular should be considered preliminary. Despite these limitations, this unique study offers valuable insights into the presence of diurnal rhythms in preterm milk and provides an opportunity to develop future prospective studies into chrononutrition in the NICU.

As evidence on 24-h variation in human milk composition grows, future research could explore whether informing lactating mothers about this topic and labelling stored milk with the time of expression provides meaningful benefits for infants. Such practices would represent a low-cost, low-burden intervention but require systemic evaluation before recommendations can be made. Currently, standard infant formula does not account for 24-h variations in milk components (49, 69, 70). Compared to formula fed infants, breastfed infants show improved sleep parameters and a more regular nocturnal increase in 6-sulfatoxymelatonin, a metabolite of melatonin (71). Moreover, Aparicio et al. (7) have shown that infants receiving a tryptophan-enriched formula at night improved sleep parameters. These observations suggest that formula-fed infants could benefit from supplementation with exogenous melatonin. However, more research on targeted nutritional strategies is needed to investigate these effects.

## 5 Conclusion

This study demonstrates consistent diurnal variations in melatonin and cortisol across all lactational stages in milk from mothers of very preterm infants, extending previous work on milk composition in very preterm human milk to hormonal rhythms (72). To our knowledge, consistent individual 24-h hormonal rhythms in very preterm human milk have not previously been characterized at this level of detail. These findings demonstrate that maternal hormonal rhythmicity is preserved in milk from the earliest stages of preterm lactation.

Clinical implications can be meaningful, given that preterm infants admitted to the NICU are routinely fed with mistimed expressed milk, and donor milk does not account for diurnal variation (35). Our study provides biological rationale for investigating time-of-day labelling of expressed milk and may warrant future research into pooling practices in human milk banks. Whether time-matched feeding could provide biologically relevant temporal signals, and whether such signals contribute to circadian entrainment or to outcomes including sleep, metabolic regulation or neurodevelopment, remains to be addressed in future prospective studies.

## Data Availability

The raw data supporting the conclusions of this article will be made available by the authors, without undue reservation.
