# Supplementary material for: Diurnal rhythms in melatonin and cortisol in very preterm human milk
Source: Front Nutr. 2026 Jun 16;13:1845043. doi: 10.3389/fnut.2026.1845043 (PMC13314464; doi:10.3389/fnut.2026.1845043)
Supplement: Supplementary file 1 [file Table_1.DOCX]

**Supplementary figures**

**
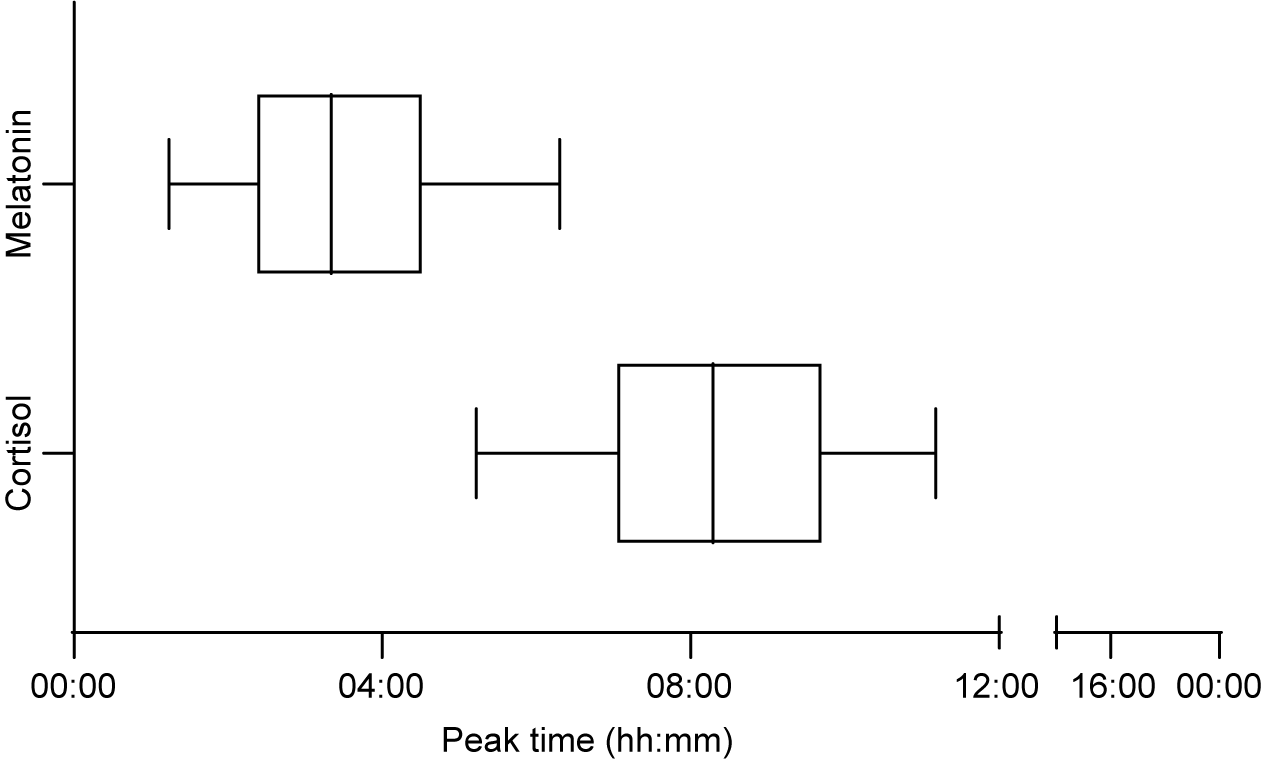
**

**Supplementary Figure 1: Boxplots showing the distribution of peak time (hh:mm) of cortisol (n=18) and melatonin (n=25).** Boxes represent the interquartile range (IQR) and median, error bars represent the minimum and maximum values. Melatonin peaks were typically observed during the early night (median ≈ 04:00 h), while cortisol peaks occurred in the morning hours (median ≈ 08:00 h), reflecting distinct di-urnal rhythms of the two hormones.

**
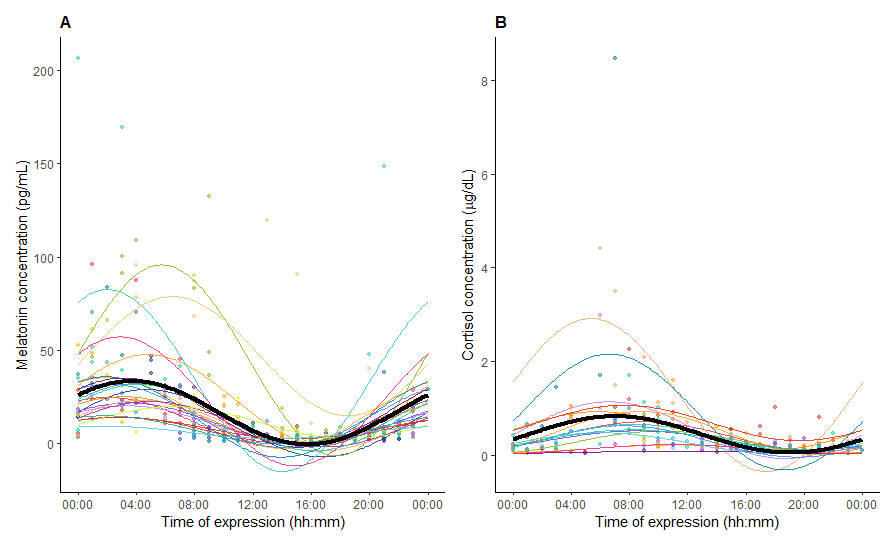
Supplementary Figure 2: Significant 24-hour rhythmicity in melatonin and cortisol levels in preterm human milk from mothers of deceased infants.** (A) Melatonin concentrations for each individual milk series exhibiting diurnal rhythmicity (n=23). (B) Cortisol concentrations for each individual milk series exhibiting diurnal rhythmicity (n=16). The y-axis represents the melatonin (pg/mL) and cortisol (µg/dL) concentration, the x-axis represents time of expression (hh:mm). Each data point represents one milk sample. Each figure shows hormone concentrations from samples collected over three consecutive days on a 24-hour scale. Each colored line and corresponding dots represent one individual milk series. The black solid line represents the average nonlinear regression fit.

**

**

**Supplementary Figure 3: Melatonin and cortisol peak times and concentrations according to lactational stage.** (A) Peak time (hours) of melatonin according to lactational stage (colostrum (n=6), transitional (n=15), mature milk (n=3)). (B) Peak time (hours) of cortisol according to lactational stage (colostrum (n=3), transitional (n=12), mature milk (n=3)). (C) Peak melatonin concentration (pg/mL) according to lactational stage. (D) Peak cortisol concentration (µg/dL) according to lactational stage. Data are presented as mean ± standard deviation (SD).



**Supplementary Figure 4:** **Melatonin and cortisol peak times and concentrations according to gestational age (GA) at birth (weeks).** (A) Peak time (hours) of melatonin by GA at birth. (B) Peak time (hours) of cortisol by GA at birth. (C) Peak melatonin concentration (pg/mL) by GA at birth. (D) Peak cortisol concentration (µg/dL) by GA at birth. Data are presented as mean ± standard deviation (SD).





**Supplementary Figure 5: Amplitude of melatonin and cortisol.** (A) Melatonin amplitude according to gestational age (GA) at birth (weeks). (B) Cortisol amplitude according to GA at birth. (C) Melatonin amplitude according to lactational stage (colostrum (n=6), transitional (n=15), mature milk (n=3)). (B) Cortisol amplitude according to lactational stage (colostrum (n=3), transitional (n=12), mature milk (n=3)). Data are presented as mean ± standard deviation (SD).

**Supplementary Table 1: Melatonin and cortisol outcomes in very preterm human milk according to lactational stage**

|  | Colostrum | Transitional | Mature |
| --- | --- | --- | --- |
| Peak time melatonin (hours (AM)) | 4.2 ± 1.76 (95% CI; 2.97-5.43) (6) | 3.36 ± 1.47 (95% CI; 2.57-4.14) (16) | 3.51 ± 2.04 (95% CI; -1.5-8.61) (3) |
| Peak time cortisol (hours (AM)) | 9.23 ± 1.63 (95% CI; 5.18-13.28) (3) | 8.39 ± 7.78 (95% CI; 7.26-9.52) (12) | 6.95 ± 2.30 (95% CI; 1.24-12.66) (3) |
| Peak concentration melatonin (pg/mL) | 60.4 ± 34.1 (95% CI; 24.5-96.2) (6) | 53.0 ± 36.7 (95% CI; 32.6-73.3) (16) | 19.1 ±10.1 (95% CI; -5.98-44.2) (3) |
| Peak concentration cortisol (µg/dL) | 0.87 ± 0.8 (95% CI; -1.1-2.9) (3) | 2.6 ± 4.76 (95% CI; -0.47-5.57) (12) | 1.64 ± 1.62 (95% CI; -2.38-5.66) (3) |
| Phase difference | 5.68 ± 2.08 (95% CI; 0.53-10.84) (3) | 5.14 ± 2.15 (95% CI; 3.78-6.50) (12) | 3.41 ± 2.16 (95% CI; -1.97-8.79) (3) |

Data are presented as mean ± SD and 95% CI. The number of milk series included in each group is shown in parentheses. Milk samples were categorized by lactational stage: colostrum (milk collected from birth (day 0) up to the fourth day of life), transitional (milk collected from day five up to the second week postpartum) or mature (collected from two weeks postpartum onwards). Abbreviations: CI; confidence interval, SD; standard deviation.

**Supplementary Table 2: Melatonin and cortisol outcomes in very preterm human milk according to infant sex**

|  | Female | Male | p-value |
| --- | --- | --- | --- |
| Peak time melatonin | 3.58 ± 1.67 (95% CI; 2.52-4.64) (12) | 3.58 ± 1.30 (95% CI; 2.80-4.37) (13) | 0.996 |
| Peak time cortisol | 7.03 ± 1.26 (95% CI; 5.98-8.09) (8) | 9.29 ± 1.66 (95% CI; 8.11-10.49) (10) | 0.0058** |
| Peak concentration melatonin (pg/mL) | 39.99 ± 30.37 (95% CI; 19.59 - 60.39) (12) | 59.57 ± 37.68 (95% CI; 36.80-82.34) (13) | 0.18 |
| Peak concentration cortisol (µg/dL) | 1.54 ± 0.93 (95% CI; 0.75-2.32) (8) | 2.59 ± 5.29 (95% CI; -1.2 -6.37) (10) | 0.59 |
| Phase difference | 4.0 ± 1.56 95% CI; (2.70-5.31) (8) | 5.69 ± 2.32 (95% CI; 4.03-7.35) (10) | 0.096 |

Data are presented as mean ± SD and 95% CI. The number of milk series included in each group is shown in parentheses. Peak times are expressed in hours (AM). P-values indicate differences between milk of mothers of female and male infants. Statistically significant differences are indicated as *p < 0.05; **p < 0.01. Abbreviations: CI; confidence interval, SD; standard deviation.
